# Transgenerational and within‐generation plasticity shape thermal performance curves

**DOI:** 10.1002/ece3.4900

**Published:** 2019-01-29

**Authors:** Grisel Cavieres, José M. Alruiz, Nadia R. Medina, José M. Bogdanovich, Francisco Bozinovic

**Affiliations:** ^1^ Center of Applied Ecology and Sustainability (CAPES), Departamento de Ecologia, Facultad de Ciencias Biológicas Pontificia Universidad Católica de Chile Santiago Chile; ^2^ CCT‐Mendoza CONICET, Grupo de Investigaciones de la Biodiversidad, CONICET Instituto Argentino de Investigaciones de Zonas Áridas Mendoza Argentina

**Keywords:** climate change, early experience, environmental temperature, thermal performance curve, transgenerational plasticity

## Abstract

Thermal performance curves (TPCs) compute the effects of temperature on the performance of ectotherms and are frequently used to predict the effect of environmental conditions and currently, climate change, on organismal vulnerability and sensitivity. Using *Drosophila melanogaster* as an animal model, we examined how different thermal environments affected the shape of the performance curve and their parameters. We measured the climbing speed as a measure of locomotor performance in adult flies and tested the ontogenetic and transgenerational effects of thermal environment on TPC shape. Parents and offspring were reared at 28 ± 0ºC (28C), 28 ± 4ºC (28V), and 30 ± 0ºC (30C). We found that both, environmental thermal variability (28V) and high temperature (30C) experienced during early ontogeny shaped the fruit fly TPC sensitivity. Flies reared at variable thermal environments shifted the TPC to the right and increased heat tolerance. Flies held at high and constant temperature exhibited lower maximum performance than flies reared at the variable thermal environment. Furthermore, these effects were extended to the next generation. The parental thermal environment had a significative effect on TPC and its parameters. Indeed, flies reared at 28V whose parents were held at a high and constant temperature (30C) had a lower heat tolerance than F1 of flies reared at 28C or 28V. Also, offspring of flies reared at variable thermal environment (28V) reached the maximum performance at a higher temperature than offspring of flies reared at 28C or 30C. Consequently, since TPC parameters are not fixed, we suggest cautiousness when using TPCs to predict the impact of climate change on natural populations.

## 1 INTRODUCTION

Climate is changing. Mean global temperatures, thermal variability, and the frequency of extreme environmental events have increased, and these have had significant impacts on biodiversity (Dawson, Jackson, House, Prentice, & Mace, 2011; Gitay, Suárez, Watson, & Dokken, 2002; Meehl & Tebaldi, 2004; Pachauri & Reisinger, 2007; Palmer et al., 2017; Vázquez, Gianoli, Morris, & Bozinovic, 2017). These changes in the thermal environment are expected to be particularly challenging for ectotherms because their physiology is directly dependent on ambient temperatures (Pörtner, 2002; Saxon, O'Brien, & Bridle, 2018; Sunday, Bates, & Dulvy, 2011). To a large extent the susceptibility and vulnerability of ectotherms to climate change has been assessed through the study of thermal performance curves (TPCs) which characterize the relationship between performance or fitness and body temperature (Sinclair et al., 2016). Indeed, TPCs have often been used to determine how ectothermic species will respond to ongoing climate change (Angilletta, 2009; Deutsch et al., 2008; Dillon, Wang, Garrity, & Huey, 2009; Estay, Lima, & Bozinovic, 2014; Huey et al., 2012). In that sense, Colinet, Sinclair, Vernon, and Renault (2015) reported that thermal variability within tolerant physiological ranges of organism improve performance. Also, Cavieres, Bogdanovich, and Bozinovic (2016) studying the effects of thermal variability in TPC indicated that flies reared in fluctuating thermal environments improved heat tolerance compared with flies from constant thermal environments. Parallelly, it has been reported that the nature of thermal variability shape TPC in ectotherms (Cavieres, Bogdanovich, Toledo, & Bozinovic, 2018; Kingsolver, Higgins, & Augustine, 2015; Kingsolver & Woods, 2016). As such, TPCs differ depending on physiological tolerance to environmental thermal conditions, and the thermal history experienced by each phenotype (Bozinovic, Medina, Alruiz, Cavieres, & Sabat, 2016; Cavieres, Nuñez‐Villegas, Bozinovic, & Sabat, 2017; Huey & Berrigan, 1996; Huey et al., 2012).

Organisms may react to environmental inputs through phenotypic plasticity (Burggren, 2018; Sultan, 2015). Plasticity is heritable and appears to evolve through natural selection (Forsman, 2015; Scheiner & Lyman, 1989). The modification of an organism by the environment is often hypothesized to be responsible for allowing organisms to adjust to changing environmental conditions through improving organismal function and fitness (Kelly, Panhuis, & Stoehr, 2012; Nunney, 2016; Nunney & Cheung, 1997). When environmental conditions change over short time scales, individuals can exhibit continuous and reversible phenotypic transformations (Piersma & Drent, 2003). During the early ontogeny, organisms are highly sensitive to environmental cues (Burggren & Mueller, 2015; Saxon et al., 2018; Spicer, Rundle, & Tills, 2011). Thus, developmental conditions can induce modifications in phenotype and potentially lead to irreversible changes (Burggren, 2018; Cooper, Tharp, Jernberg, & Angilletta, 2012; Dufty, Clobert, & Møller, 2002). In that sense, Cavieres et al. (2017) studying the putative effects of early life experience on physiological plasticity, reported an ontogenetic dependence of plastic response in rodents. That is, environmental conditions experienced during the development determined the ability to modify the phenotype during adulthood (see Weinig and Delph (2001)). Moreover, the plastic response is a property of the trait, not the individual and may be constrained by the costs of maintenance and production of plastic structures (DeWitt, Sih, & Wilson, 1998; Gilbert & Epel, 2008; Pigliucci, 2001; Sultan, 2015).

Phenotypic changes in early ontogeny have long‐term implications on an organism's performance (Jablonka & Raz, 2009; Mousseau & Dingle, 1991), and may persist over generations despite a lack of alterations in gene sequences (Badyaev & Uller, 2009; Ho & Burggren, 2010). The transgenerational transfer also called parental effects (Badyaev & Uller, 2009), transgenerational plasticity (Marshall & Uller, 2007) and transgenerational memory (Molinier, Ries, Zipfel, & Hohn, 2006) is used to describe the transmission of traits, factors, and/or information that induces phenotypic changes from one generation to the next (Ho & Burggren, 2010). Such effects could enable offspring receive information early during the development and modify the phenotype adaptatively according to parental information to best respond to their environment (Engqvist & Reinhold, 2016; Klosin, Casas, Hidalgo‐Carcedo, Vavouri, & Lehner, 2017; Mousseau & Fox, 1998; Salinas, Brown, Mangel, & Munch, 2013; Schmalhausen, 1938; Young & Badyaev, 2007). For instance, Rodríguez‐Romero, Jarrold, Massamba‐N'Siala, Spicer, and Calosi (2016) reported that transgenerational plasticity drove the increase of reproductive output in the marine polychaete *Ophryotrocha labronica* after three generations under low pCO2 conditions. Also, Crill, Huey, and Gilchrist (1996) have found that the ambient temperature experienced by parents influences heat tolerance in the fruit fly *D. melanogaster*. Thus, transgenerational transfer appears as a valuable source of variation between individuals, influencing short‐term selection and the evolutionary trajectory of a population (Bonduriansky, Crean, & Day, 2012; Mousseau & Dingle, 1991; Rodríguez‐Romero et al., 2016; Young & Badyaev, 2007). Overall, the transgenerational transfer may depend on the physiological status of parents, duration of exposure, and environmental signal (Burggren, 2014; Donelson, Wong, Booth, & Munday, 2016).

Studies of the effects of rapid environmental changes are normally based on its direct effects on organisms, minimizing the potential transgenerational ecological and evolutionary effects. Transgenerational effects on organisms may reveal the mechanisms through which populations could diminish the effects of climate change. Specifically, transgenerational impacts on thermal performance may have important implications on life‐history processes since may alter the extinction risk posed by changing climate as demonstrated by Salinas and Munch (2012). Indeed, Sales et al. (2018) showed in beattles that heat waves impact populations across generations, which highlight the importance of seeing transgenerational effects when estimating ecological and evolutionary effects on organisms. Consequently, here we tested the effect of different environmental thermal regimes (constant and variable conditions) on organismal performance, measured as locomotor performance through climbing speed records in the fruit fly *Drosophila melanogaster*. Specifically, we assessed how early life exposure to thermal regime affected (or not) the TPC of one generation of adults flies as well as of a subsequent generation. Thus, we studied the entire performance curve and, we estimated their parameters to compare individuals. Many studies have documented thermal acclimation on thermal limits, but few have measured the effects on the entire performance curve, and to the best of our knowledge, none have looked at transgenerational effects on TPCs. Overall, we hypothesized that flies experiencing high temperatures and variable environments during development would have increased thermal tolerance, and their TPCs would be shifted to the right. Also, we hypothesized that the offspring of parents in the high temperature and variable temperature treatments would have increased thermal tolerance compared to controls despite not having directly experienced these conditions.

## 2 MATERIALS AND METHODS

The fruit flies, *Drosophila melanogaster,* were used as animal model. Previously we have used this species to test hypotheses regarding the effects of thermal variability on performance and fitness (Bozinovic, Catalan, Estay, & Sabat, 2013; Bozinovic et al., 2016; Cavieres et al., 2016). Moreover, the phenotypic responses of this organism to environmental temperature and other climatic factors are well known (Bozinovic et al., 2011; Hoffmann, 2010; Ragland & Kingsolver, 2008).

**Figure 1:**
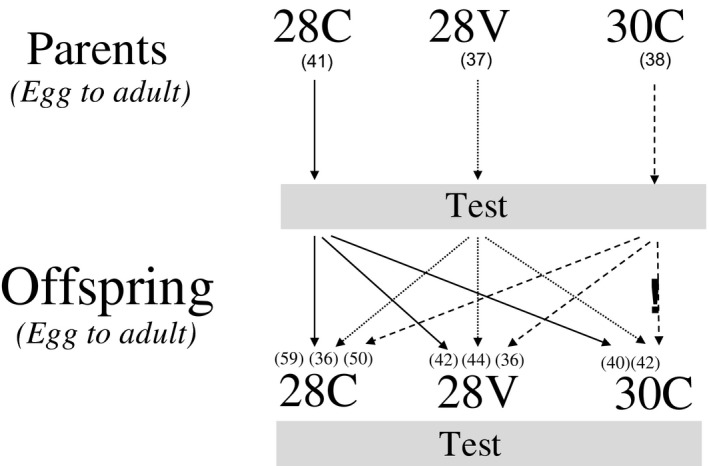
Experimental design to assess the ontogenetic and transgenerational effects of thermal environment on thermal performance curve shape. Parent and offspring generations were acclimated in one of three different thermal environments 28 ± 0ºC (28C), 28 ± 4ºC (28V), and 30 ± 0ºC (30C). Eggs from flies reared at 30C did not hatch at 30C. Numbers in parentheses are sample size

Adult *D. melanogaster* were collected in central Chile (33°26 S; 70°39 W at 500 m above sea level) during summer 2016. Flies were identified based on morphological characters (Markow & O'Grady, 2005). After collection, ten breeding groups were made; each group consisted of approximately 10 males and 10 females. Groups were reared in controlled conditions at 24°C and L:D = 12:12. Flies were grown in 250‐ml glass vials with Burdick culture medium (Burdick, 1955) to constituting the stock bottles. Third generation adult flies from the stock were randomly assigned to one of three thermal treatments set based on the limits of fruit fly egg viability (eggs‐to‐adult viability is near 80% at 28ºC and 0%–5% at 32ºC, for details see Hoffmann, 2010; Cavieres et al., 2018): (a) moderate mean and no variance (28 ± 0°C, “28C”), (b) moderate mean and high variance (28 ± 4°C, “28V”), and (c) high mean and no variance (30 ± 0°C, “30C”). Flies were maintained in each treatment in climatic chambers (PITEC, Model BIOREF) from eggs to adult; then, breeding groups were distributed among the three treatments to obtain F1 (Figure 1). In the 28V treatment, the temperature increased linearly, reached a maximum of 32°C, remained constant, and then decreased until a minimum temperature of 24°C was reached. The heating/cooling rate between the minimum and maximum temperatures was 0.03°C/min.

We quantified the effect of temperature on locomotor performance, measured as climbing speed of adults from the parental and offspring generations. Speed is often used as a proxy of organismal performance because it is correlated with mating success (Gibert, Huey, & Gilchrist, 2001; Gilchrist, Huey, & Partridge, 1997). Specifically, we quantified wall climbing speed (cm/s) by knocking down a fly in a narrow glass test tube (12 × 100 mm) and measuring the time required to walk up the tube to a height of 7.0 cm. Each fly was kept for 10 min at the test temperature. Each fly was tested at the following temperatures: 16, 20, 24, 28, 32, 36, 38, 39 and 40°C (see (Cavieres et al., 2016). All animals remained in a room at 24 ± 2°C between tests (around 10 min). A third‐order polynomial function was fit to the Thermal performance curve, and its parameters were estimated: the upper and lower thermal limits (CT_max_ and CT_min_, respectively), maximum performance (*V*_max_), performance breadth (*T*_br_) according to (Gilchrist, 1996), and the temperature at which performance was maximized (*T*_o_).

### 2.1 Statistical analyses

To quantify effects of temperature on climbing speed, we fit a third‐degree polynomial function for the entire TPC and performed a linear mixed model to test the impact of mean and variance of temperature TPC during ontogeny as well as in the next generation. The linear mixed model was generated for the longitudinal data; individuals (random intercept) were nested in temperature (slope) and included as a random effect.

Also, we assessed the effects of temperature on TPC parameters. We test the variables using a linear model when variables follow a normal distribution and a general linear model when the variables follow a different distribution. To test the effects of temperature during ontogeny we used *thermal treatment* as a factor, and to examine the transgenerational effects we used Parental thermal treatment*Offspring thermal treatment as factors. Multiple comparisons were restricted sets of contrasts among offspring reared at the same thermal environment. False Discovery Rate (FDR) correction for multiple comparisons was applied.

To estimate the parameters of each TPC, we used the GitHub R package *ThermPerf*. All analyses were carried out using R (http://www.R-project.org/).

## 3 RESULTS

**Figure 2:**
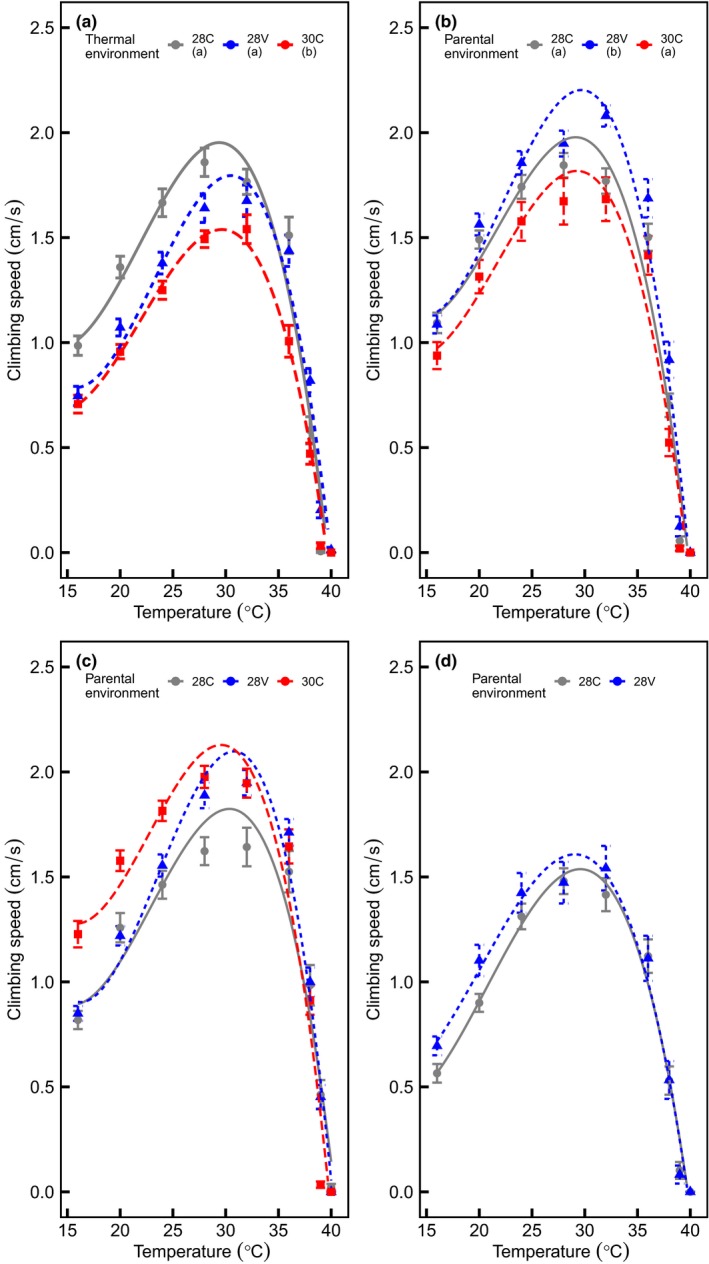
Ontogenetic and Transgenerational effects of temperature on thermal performance curve. Offspring and parents were reared in one of three environments 28 ± 0ºC (28C), 28 ± 4ºC (28V), and 30 ± 0ºC (30C). Thermal performance curves were fit to a third‐order polynomial function. (a) Ontogeny, climbing speed of flies reared at 28C, 28V, and 30C. (b‐d). Transgenerational effects of the thermal environment. The climbing speed of flies reared at 28C, 28V, and 30C respectively. Eggs from flies reared at 30C did not hatch at 30C. Shapes and colors indicate the thermal environment experienced by of parental population. Different letters indicate a significant difference between groups

As expected, the locomotor performance was significantly affected by temperature (Table 1, Figure 2). High mean temperature and thermal variability shaped the TPC of *D. melanogaster*. Thermal performance was lower in flies reared at 30C than those reared at 28C and 28V (Table 1 and Figure 2a). Indeed, flies reared at 30C, exhibited lower *V*_max_ and *Tbr* than flies reared at 28C and 28V. Flies developed in variable thermal conditions improved heat tolerance, that is, increased CT_min_, CT_max_ *,* and *To* in comparison to flies reared at 28C (Table 1).

**Table 1:** Coefficients of the linear mixed model fitted to data for climbing speed in *Drosophila melanogaster* acclimated during ontogeny in one of three thermal environments: 28 ± 0ºC (28C), 28 ± 4ºC (28V), and 30 ± 0ºC (30C). The entire thermal performance curve (A) and its parameters (B). Maximum performance (*V*_max_), optimum temperature (*T*_o_), performance breadth (*T*_br_), critical thermal maxima (CT_max_), and critical thermal minima (CT_min_). Values for Significant values are indicated in bold (*p < 0.05*)

Effect	Coefficient	*SE*	*T*	*p*	
(A) Entire thermal performance curve					
Intercept (28C)	1.09	0.03	31.78	**<0.001**	
28V	−0.04	0.05	−0.71	0.48	
30C	−0.25	0.05	−5.10	**<0.001**	
(B) Performance curve parameters					
*V*_max_ (cm/s)					
Intercept (28C)	2.06	0.03	22.7	**<0.001**	
28V	−0.13	0.03	−1.36	0.17	
30C	−1.02	0.03	−5.46	**<0.001**	
*To* (ºC)					
Intercept (28C)	29.14	0.24	118.9	**<0.001**	
28V	1.35	0.36	3.72	**0.001**	
30C	0.21	0.36	1.21	0.22	
*T*_br_ (ºC)					
Intercept (28C)	11.53	0.23	49.88	**<0.001**	
28V	0.03	0.34	0.08	0.94	
30C	−0.81	0.33	−2.44	**0.01**	
CT_max_ (ºC)					
Intercept (28C)	39.11	0.06	631	**<0.001**	
28V	0.44	0.09	4.67	**<0.001**	
30C	−0.08	0.09	−0.89	0.37	
CT_min_ (ºC)					
Intercept (28C)	13.1	0.20	64.7	**<0.001**	
28V	0.60	0.29	2.03	**0.04**	
30C	0.25	0.29	0.88	0.38	

**Figure 3:**
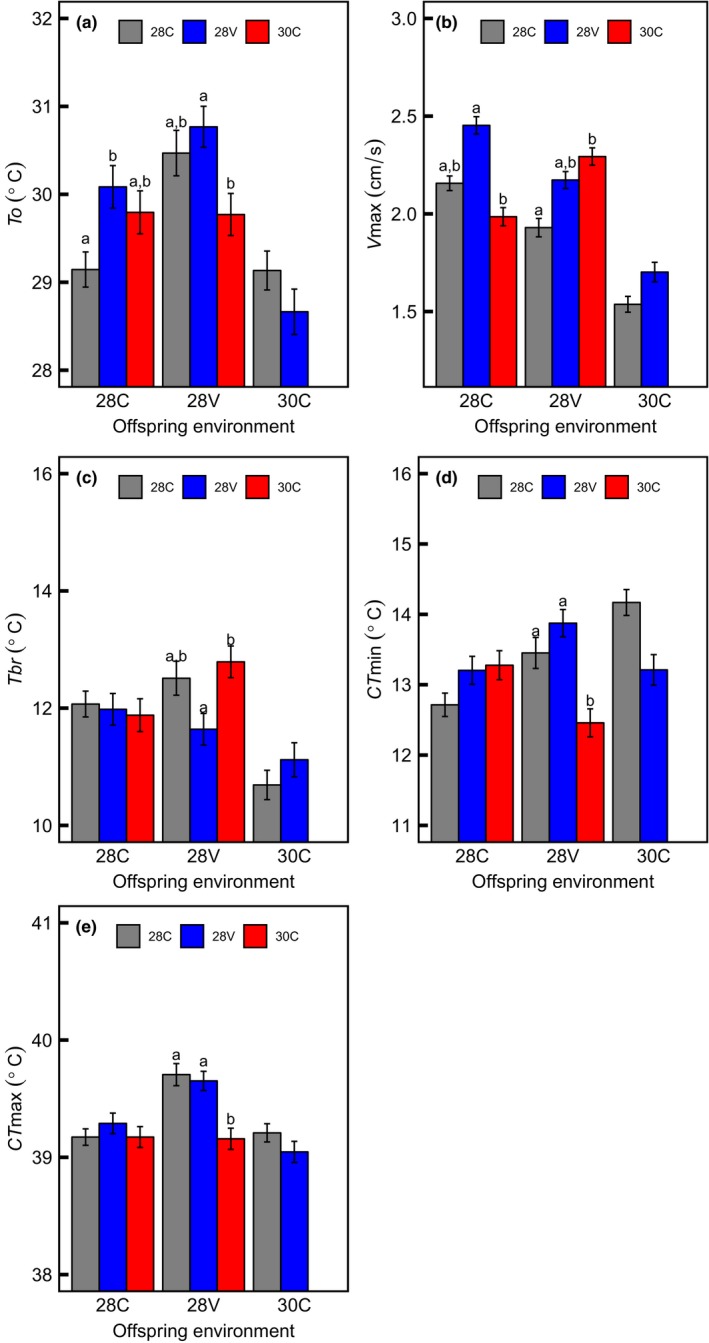
Transgenerational effects of temperature on the performance curve parameters estimated from a third‐order polynomial fit. Y‐axis is offspring performance (*V*_max_, *To*, *Tbr*, CT_max_, and CT_min_), and the X‐axis is the thermal environment in which the offspring were raised. Bars indicate the response when parents were maintained in one of three different environments: 28 ± 0ºC (28C), 28 ± 4ºC (28V), and 30 ± 0ºC (30C). Eggs from flies reared at 30C did not hatch at 30C. Different letters indicate a significant difference between groups

The effects of thermal conditions experienced during ontogeny were extended to the subsequent generation (Figure 2b and Figure 3). We found a significative interaction between parental and offspring thermal environment on the entire TPC (Table 2) and its parameters (Figure 3). In an environment with moderate mean and no variance (28C), the thermal performance (i.e., the entire TPC) was higher in offspring from flies reared at 28V than offspring of flies held at 30 and 28C (Figure 2b). The analyses of thermal performance parameters revealed that this increase was due to an increase in *V*_max_ (Figure 2b). Flies reared at variable thermal environments whose parents were held at high and constant temperature (i.e., 30C) decreased heat tolerance, that is, had lower thermal limits than offspring of flies reared at 28C and 28V, shifting the TPC to the left (Figure 3d,e), with a lower To than offspring of flies reared at 28V (Figure 3 A respectively). Moreover, offspring from flies reared at 28V but living at 28C reached the maximum performance (*V*_max_) at a higher *To* than offspring of flies reared at 28C. We did not find transgenerational effects of temperature on flies reared at 30C (Figures 2 and 3).

**Table 2:** Coefficients of the linear mixed model fitted to the entire thermal performance curve of Offspring in *Drosophila melanogaster* acclimated to one of three environments (28C, 28V and 30C). Thermal environment of parents in parentheses. Significant values are indicated in bold (p < 0.05) (for multiple comparisons see Figure 2)

Effect	Coefficient	*SE*	*df*	*T*	*p*
Intercept (Offspring 28C (Parents 28C))	1.15	0.04	2804	29.35	**<0.001**
Offspring 28C (Parents 27V)	0.17	0.06	343	2.83	**0.004**
Offspring 28C (Parents 30C)	−0.08	0.06	343	−1.38	0.17
Offspring 28V (Parents 28V)	0.07	0.06	343	1.14	0.25
Offspring 28V (Parents 28V)	0.13	0.05	343	2.26	**0.02**
Offspring 28V (Parents 30C)	0.11	0.06	343	1.78	0.07
Offspring 30C (Parents 28C)	−0.24	0.05	343	−4.28	**<0.001**
Offspring 30C (Parents 28V)	−0.22	0.06	343	−3.49	**<0.001**

## 4 DISCUSSION

Our key finding was that the environmental thermal variability and high and constant temperature experienced during early ontogeny shape the thermal performance curve. Also, those effects are extended to the next generation but depending on the thermal environment in which offspring live. High and constant environmental temperature reduced the thermal performance of both parental and offspring generation. The thermal variability improved the heat tolerance of parents and increased thermal performance of F1 held at a constant thermal environment. Finally, in an extreme environment, the thermal experience of parents did not affect offspring's performance.

Environmental variability through time imposes selection pressure (Gould, 1985), thus driving adaptation to different thermal environments (Kubrak, Nylin, Flatt, Nässel, & Leimar, 2017; Sultan, 2015; Young & Badyaev, 2007). The ability of organisms to produce different phenotypes under changing environmental conditions is influenced by environmental signals and the temporal window at which signals occur (Burggren & Mueller, 2015; Burggren & Reyna, 2011; Spicer et al., 2011). Here, we showed that flies experiencing environmental thermal variability during ontogeny improved heat tolerance, through changes in CT_max_ *, *CT_min_ *, and To* values in comparison to flies that did not experience thermal variability during development (Figure 2a, Table 1). In that sense, experimental studies have shown the benefits of thermal variability on developmental time (Ragland & Kingsolver, 2008), survival (Javal, Renault, & Colinet, 2016), and population dynamics (Clavijo‐Baquet et al., 2014; Estay, Clavijo‐Baquet, Lima, & Bozinovic, 2011). Brief exposure to high temperatures may induce the expression of stress‐inducible heat‐shock proteins (HSPs) and increase thermal tolerance in ectotherms exposed to extreme temperature or thermal variability (Colinet et al., 2015; Dong, Miller, Sanders, & Somero, 2008; Lewis et al., 2016; Tomanek, 2010). Additionally, it has been proposed that the increased performance in variable environments may be explained by to recovery time between extreme events, which enable periodic opportunities to return to physiological homeostasis (Colinet et al., 2016).

On the other hand, it has been reported a high cost of living in extreme environments (DeWitt et al., 1998; Kafri, Metzl‐Raz, Jona, & Barkai, 2016; Pigliucci, 2001). Prolonged exposure to extreme temperatures has adverse effects on fitness, reducing survival and rate of development (Chown & Terblanche, 2006; Colinet et al., 2016; Feder & Hofmann, 1999; Krebs & Feder, 1997). Indeed, we observed that in flies exposed to 30C during ontogeny, *V*_max_ was reduced, and their offspring not hatched at 30C (Figure 1, see also Hoffmann 2010). Contrary to our predictions, offspring from 30C flies reared at 28V reduced heat tolerance in comparison to the progeny of 28C and 28V. Thus, the elevated cost of living in an extreme thermal environment reduce thermal tolerances during ontogeny (see Table 1 and Figure 2a), and on the subsequent generation (see Figure 3). Despite that studies on nongenetic inheritance mechanisms across multiple generations have increased (Donelson et al., 2016; Shama et al., 2016; Thor & Dupont, 2015), it is still unclear the impact and duration of transgenerational effects (see Burggren, 2015). Phenotypic plasticity during early ontogeny may induce the emergence of new phenotypes (Bartheld, Artacho, & Bacigalupe, 2017; Cavieres et al., 2016, 2017; Kingsolver et al., 2015; Koussoroplis, Pincebourde, & Wacker, 2017). In flies reared at 28V, more heat tolerant phenotypes. Despite the increase of thermal performance in flies reared at 28V, the cost of phenotypic plasticity here is unknown (not assessed in this study). Meats (2011) studying the thermal tolerance in Queensland fruit fly reared in regimes of variable and constant temperature, reported that the increased in thermal tolerance in one stage of development affected negatively the survival rate during the next stage. (see also, Messenger & Flitters, 1958; Marshall & Sinclair, 2010). Besides, Folguera et al. (2011) reported that the increase of thermal amplitude affected negatively life‐history traits (longevity and growth rate), increasing metabolic cost and stress responses (synthesis of heat‐shock proteins).

Thermal conditions experienced during early life affected the next generation; in this vein, the *adaptive transgenerational plasticity hypothesis* posits that offspring reared in the same environment of their parents will have higher fitness than offspring reared in an environment different from that of their parents (Gilchrist & Huey, 2001) showed in fruit fly that F1 of parents reared at high temperatures, exhibit higher fitness independently of the thermal environment experienced. The analyses of the entire TPC showed that flies from 28C, whose parents were reared at 28V, exhibited higher performance than F1 from flies reared in 28C or 30C (Figure 2b). Besides, in a variable environment, the offspring of flies reared in 28V and 28C increased *T*_o_, CT_min_ and CT_max_ compared with F1 from flies held in 30C (Figure 3). Accordingly, although our results support the transgenerational effects of temperature, it does not support the adaptative transgenerational plasticity hypothesis (see Leroi, Bennett, & Lenski 1994).

The parental experience could result in “pre‐adapted” (sensu* lato*) progeny that exhibits traits that allow them to respond to the environment's challenges accurate (Engqvist & Reinhold, 2016; Salinas et al., 2013). Thus, transgenerational effects can impact mating success and reproduction (Morimoto, Simpson, & Ponton, 2017)*, *thermal preference (So & Schwanz, 2018)*,* growth (Salinas & Munch, 2012)*, *among others (see Molinier et al., 2006; Herman & Sultan, 2011; Shama et al., 2016; Donelson, Salinas, Munday, & Shama, 2017). To the best of our knowledge, this is the first study that tested the transgenerational effects of variable thermal environments on animals thermal performance. We showed that early life exposure to thermal variability, and extreme temperature shapes the TPCs of the fruit fly, and interestingly, these effects hold to the next generation. These results highlight the importance of incorporating ontogenetic and transgenerational effects of temperature in physiological studies to building robust predictions about the impact rapid environmental thermal fluctuations, changes in mean temperature or the effects of extreme thermal events on animal performance, and to avoid underestimating the potential plastic response of organisms. Consequently, we must consider how this might impair our ability to accurately predict the impact of climate change on natural populations when using TPCs.

## CONFLICT OF INTEREST

The authors declare no conflict of interest.

## AUTHOR CONTRIBUTIONS

G.C and F.B designed the experiment. N.M, and J.M.A. performed the experiments, G.C. and J.M.B analyzed the data, G.C and F.B. wrote the paper.

## Data Availability

The datasets analyzed during the current study will be available in the Dryad Digital Repository.
